# Orthodontic Treatment Need and Its Relation to Gender, Oral Hygiene, and Age Range in a Mexican Population

**DOI:** 10.7759/cureus.75088

**Published:** 2024-12-04

**Authors:** María Belén Avila Carranza, Adriana Torre Delgadillo, Alan Martínez Zumarán, Miguel Ángel Noyola Frías, Pedro Rosales García, Nuria Patiño-Marín, Marco Felipe Salas Orozco

**Affiliations:** 1 Orthodontics and Dentomaxillofacial Orthopedics, Faculty of Stomatology, Autonomous University of San Luis Potosí, San Luis Potosí, MEX; 2 Faculty of Stomatology, Northeastern Regional Complex, Meritorious Autonomous University of Puebla, Puebla, MEX; 3 Clinical Research Laboratory, Faculty of Stomatology, Autonomous University of San Luis Potosí, San Luis Potosí, MEX

**Keywords:** dental aesthetic index, gender differences, malocclusion, mexican population, orthodontic treatment need

## Abstract

Introduction

Malocclusion, a prevalent oral health concern, significantly impacts both functional abilities and psychosocial well-being. The Dental Aesthetic Index (DAI) is widely used to assess malocclusion severity and orthodontic treatment needs. This study aims to evaluate the prevalence of orthodontic treatment needs based on the DAI in a Mexican population, considering factors such as gender, oral hygiene, and age range.

Materials and methods

This retrospective observational study analyzed 639 patient records from the Orthodontics and Dentomaxillofacial Orthopedics postgraduate program at the Autonomous University of San Luis Potosí, San Luis Potosí, Mexico. The DAI was used to assess malocclusion severity, and patients were classified into four categories based on their scores. Data on gender, oral hygiene, and age were also collected and analyzed.

Results

The results showed no significant association between DAI levels and gender, oral hygiene, or age range. However, a significant association was found between missing teeth and higher DAI scores (OR = 8.9), maxillary irregularity (OR = 8.6), and open bite (OR = 4.9). The prevalence of severe malocclusion (DAI ≥ 36) was notably high, affecting over 50% of the study population.

Discussion

The findings align with previous studies that highlight the influence of structural irregularities such as open bite and maxillary irregularities on severe malocclusion. While no significant associations were found with gender, oral hygiene, or age, the high prevalence of severe malocclusion underscores the need for early orthodontic intervention in Mexican populations.

Conclusion

This study demonstrates a high prevalence of severe malocclusion in a Mexican population and highlights the importance of addressing structural dental irregularities to prevent long-term complications. These findings emphasize the need for accessible orthodontic care and early screening programs.

## Introduction

Globally, malocclusion is often considered the third most critical oral health concern due to its widespread occurrence and its impact on both functional abilities and mental and social health [1]. Issues arising from malocclusion include challenges with chewing and speech, temporomandibular joint (TMJ) disorders, and changes in dental aesthetics that can influence psychological well-being, self-perception, and social interactions [2]. While various indices can measure the severity of malocclusion, the Dental Aesthetic Index (DAI), established in 1986, is one of the most used in epidemiological research for determining the prevalence of malocclusion and the need for orthodontic treatments. This was confirmed when the World Health Organization (WHO) adopted the DAI as an international index to evaluate malocclusion [3].

The DAI employs a mathematical formula that calculates a score based on measurements related to malocclusion (such as spacing, molar classification, open bite, crowding, irregularity, overjet, and missing teeth) [4]. It stands out for its reliability, objectivity, simplicity, and ability to provide extensive information regarding the type and intensity of malocclusion. Despite some limitations of the DAI formula, such as the potential underestimation of deep and crossbite malocclusions, its clinical and research utility makes it valuable. The DAI is crucial not only in gauging the severity of malocclusion and its oral health implications but also in prioritizing treatment needs [5].

Recent studies have highlighted the importance of identifying factors such as gender, oral hygiene habits, and age in understanding the variation in orthodontic treatment requirements across different populations. Gender differences in malocclusion severity and oral hygiene practices have been documented, yet the findings remain inconsistent across various studies and regions [6]. Similarly, age is a critical factor, as orthodontic needs may fluctuate with the developmental stages of the dentition and skeletal structures [7]. Understanding these factors is vital for planning effective public health interventions and prioritizing orthodontic care in underserved populations [8].

Therefore, the primary objective of this study was to evaluate the prevalence of orthodontic treatment needs using the DAI in a Mexican population. The secondary objectives were to explore relevant demographic and clinical differences in these treatment needs.

## Materials and methods

This study was designed as a retrospective case-control study, utilizing the complete set of available patient records from all active patients enrolled in the Orthodontics and Dentomaxillofacial Orthopedics postgraduate program at the Autonomous University of San Luis Potosí, San Luis Potosí, Mexico, collected between January and July 2024. In the study, a sample size calculation was not performed; instead, all active patient records available at the clinic were utilized. This approach ensured the comprehensive inclusion of all relevant cases, providing a broad and representative dataset for the evaluation. The DAI has been employed in this study to assess the orthodontic treatment needs of the population due to its robust epidemiological utility and global validation. The DAI is widely recognized for its objectivity and simplicity, which facilitates standardized assessments of malocclusion severity across different populations. Its inclusion in global health studies is endorsed by the WHO, making it a reliable tool for comparing orthodontic needs across various demographic and geographic contexts. The DAI was assessed by two trained orthodontists within our postgraduate program. Each orthodontist, unaware of the other's evaluations, independently applied the DAI to classify 50 patient records randomly selected from a clinical archive. To quantify the agreement between the two evaluators, Cohen's kappa coefficient was used, resulting in a value of 0.85. This outcome indicates good agreement, demonstrating a high level of consistency between the orthodontists.

The inclusion criteria for our study were patients of both genders with complete clinical histories and the necessary data for the DAI. This included initial photographs and/or radiographs and orthodontic study models. We excluded patients without complete clinical histories, orthodontic study models, radiographs, or photographs. The population was divided into three age ranges: childhood (7-12 years), adolescence (13-18 years), and adulthood (19-62 years).

The dental hygiene of the patients was determined based on what was reported in the clinical history according to the following parameters: good (regular tooth brushing at least twice a day, daily use of dental floss, and regular visits to the dentist), regular (tooth brushing at least once a day, occasional dental floss use, and sporadic dentist visits), and bad (irregular or infrequent tooth brushing, rarely or never using dental floss, and a lack of regular dental visits).

The DAI incorporates the following components [9]: 1) the count of missing incisors, canines, and premolars in both the upper and lower jaws, 2) crowding in either 0, 1, or 2 segments (upper and lower), 3) spacing in the upper or lower jaw (0, 1, or 2), 4) measurement of diastema in millimeters between upper incisors, 5) the greatest irregularity in the upper jaw measured in millimeters, 6) the greatest irregularity in the lower jaw also measured in millimeters, 7) upper jaw overjet, 8) lower jaw overjet, 9) open bite presence, and 10) anteroposterior molar relationship, with 0 indicating normal, 1 indicating the lower first molar on any side being half a cusp mesial or distal to the upper first molar, and 2 indicating the lower first molar on any side being one or more cusps mesial or distal to the upper first molar.

The index features 10 variations corresponding to 10 occlusal ranges that are used to assess the patient’s dental condition; the scores for these ranges are multiplied by their respective weighting coefficient, yielding 10 new values which are then summed. To this sum, a constant value of 13 is added. This calculation produces the final score, which determines the patient's orthodontic treatment needs according to the four categories of the index (Table 1).

**Table 1:** Standard DAI scoring table. DAI, Dental Aesthetic Index

DAI component	Rounded weight
1. Number of missing visible teeth (incisors, canines, and premolars in the maxillary and mandibular arch)	6
2. Crowding in incisal segment (0 = no segments crowded, 1 = 1 segment crowded, 2 = 2 segments crowded)	1
3. Spacing in incisal segment (0 = no spacing, 1 = 1 segment spaced, 2 = 2 segments spaced)	1
4. Midline diastema, in millimeters	3
5. Largest anterior maxillary irregularity, in millimeters	1
6. Largest anterior mandibular irregularity, in millimeters	1
7. Anterior maxillary overjet, in millimeters	2
8. Anterior mandibular overjet, in millimeters	4
9. Vertical anterior open bite, in millimeters	4
10. Anteroposterior molar relationship, largest deviation from normal either left or right (0 = normal, 1 = 1⁄2 cusp mesial or distal, 2 = 1 full cusp or more mesial or distal)	3
11. Constant	13
Total	DAI score

**Figure 1:**
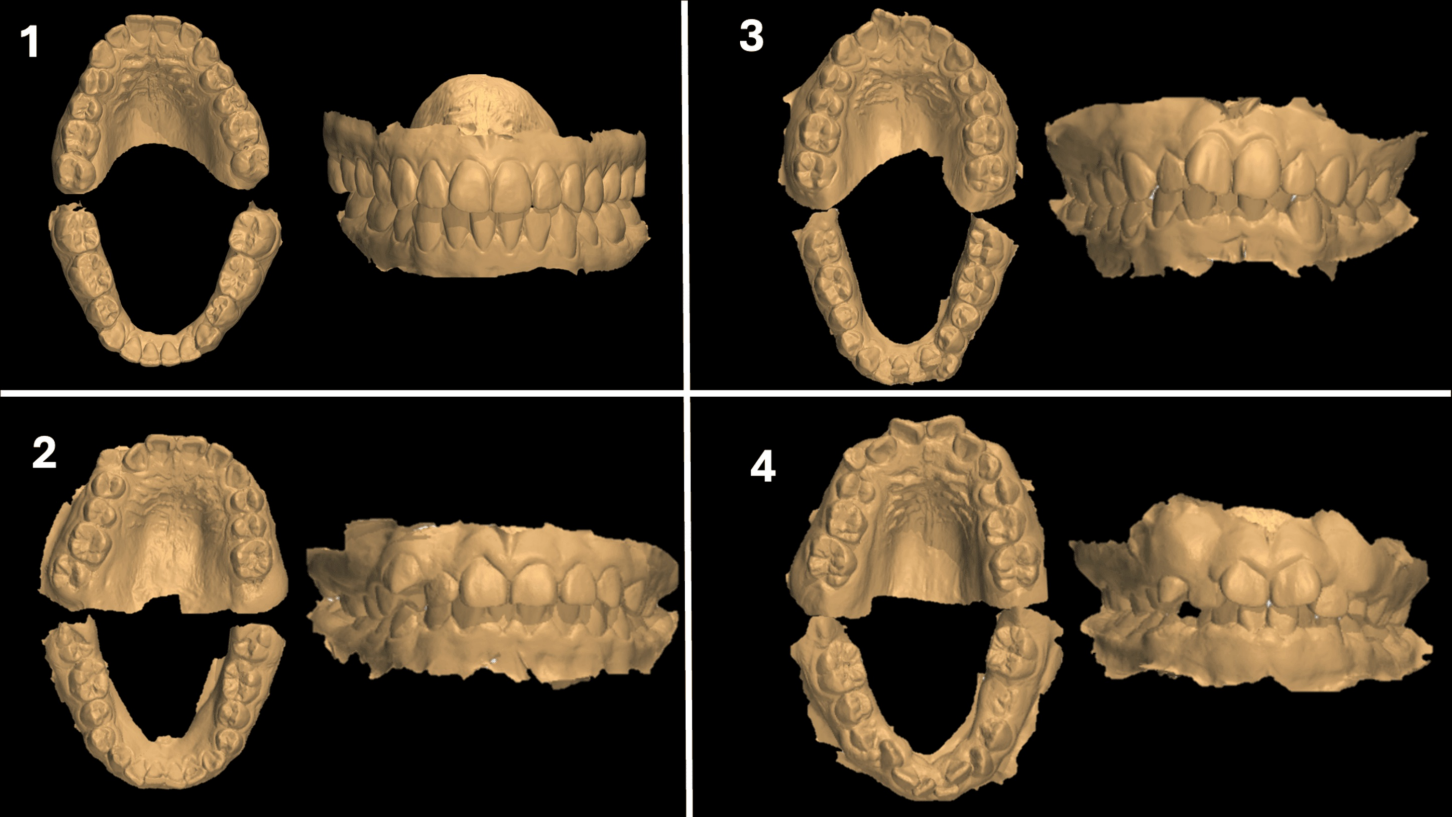
Visual examples of orthodontic treatment necessity according to DAI levels Model 1 displays minimal crowding and spacing, suggesting a DAI level 1 (score: 13) with little or no need for treatment. Model 2 shows slight misalignments and moderate crowding, particularly in the lower arch, indicative of a DAI level 2 (score: 29), where elective treatment might be considered. Model 3 presents more severe crowding and misalignment in both arches, classified as DAI level 3 (score: 33), denoting a definite need for orthodontic intervention. Lastly, model 4, with significant and pronounced malocclusion issues, including both crowding and spacing, falls into the very high DAI level 4 (score: 41), making orthodontic treatment mandatory. DAI, Dental Aesthetic Index

The DAI was used to classify patients into four levels: DAI 1 (normal or minimal malocclusion and treatment need, score ≤ 25), DAI 2 (mild malocclusion and elective treatment need, score 26-30), DAI 3 (severe malocclusion, and definite treatment need, score 31-35), and DAI 4 (incapacitating malocclusion and severe treatment need, score ≥ 36). For this study, patients were grouped into two categories: cases, which included individuals with severe or very severe malocclusions (DAI 3 and DAI 4), and controls, comprising those with minimal or moderate malocclusions (DAI 1 and DAI 2) (Figure 1).

This project received approval in January 2024 from the Ethics in Research Committee of the Faculty of Stomatology, Autonomous University of San Luis Potosí, registered under the National Commission of Bioethics with the code CEI-FE-086-022. It was unanimously approved with the following code: CONBIOÉTICA-24-CEI-001-20190213. The study was classified as no-risk research as per the guidelines set forth by the General Health Law regarding research. The information was recorded using Google Forms, and, subsequently, the database was exported to Excel for further statistical analysis.

Statistical analysis

The univariate analysis revealed that continuous variables did not follow a normal distribution, as indicated by the Shapiro-Wilk test. For these variables, non-parametric tests including the Kruskal-Wallis test for comparing multiple groups, the Mann-Whitney U test for analyzing differences between two groups, and Spearman's Rho test for assessing correlations were utilized. For categorical variables, the chi-square (χ²) test was employed to analyze differences among groups. The Levene and Brown-Forsythe tests were also used to assess the homogeneity of variances among both normally and non-normally distributed data.

A single binary logistic regression analysis was performed, where malocclusion severity was the single dependent variable. This variable was dichotomized into two subcategories based on the DAI: group 1 (DAI 1 and DAI 2) represented minimal or moderate malocclusion, while group 2 (DAI 3 and DAI 4) represented severe or very severe malocclusion. This dichotomization allowed the analysis to focus on the factors distinguishing less severe malocclusions from those requiring more urgent or intensive orthodontic intervention.

Independent variables included missing teeth, maxillary irregularity, mandibular irregularity, open bite, diastema, crowding, spacing, maxillary and mandibular overjet, molar relationship, gender, and age. Variance inflation factor analysis was conducted to detect and address multicollinearity among the independent variables. The specification error test was used to verify the assumption that the logit of the response variable is a linear combination of the independent variables. Interaction terms were tested after establishing the main effects, but none was statistically significant. The model's overall fit was assessed using the goodness-of-fit test. The association between dependent and independent variables is presented as odds ratios (OR) with 95% confidence intervals (CI). Statistical significance was set at p-values < 0.05. Data were analyzed using JMP version 9.0 (SAS Institute, Cary, NC, USA) and Stata version 11.0 (StataCorp, College Station, TX, USA).

## Results

A total of 639 records were analyzed, of which 398 (62.28%) were women and 241 (37.72%) were men. The main findings of Table 2 indicate that there were no statistically significant differences in malocclusion severity between males and females, with both genders showing similar distributions across the DAI categories. Approximately 46% of females and 29% of males had severe malocclusion (DAI ≥ 36). Additionally, the study found no significant association between DAI levels and age groups (children, adolescents, and adults), suggesting that malocclusion severity is consistent across different ages. Oral hygiene did not appear to influence the DAI scores, as no significant correlation was observed between hygiene levels and the severity of malocclusion. However, the prevalence of open bite was notably higher in females (8%) compared to males (3%), a statistically significant difference (p = 0.0134). Lastly, there were no significant gender differences in the prevalence of missing teeth, crowding, or diastema, indicating that these occlusal traits are equally distributed across the population.

**Table 2:** Association between gender with DAI esthetic and occlusal characteristics Categorical variables are presented as frequencies and percentages, while continuous variables are shown as means ± standard deviation, along with p-values to assess statistical significance between sexes. The Mann-Whitney U test and chi-square test were used to assess the significance of differences between groups. DAI, Dental Aesthetic Index

Variables n = 639	Female, n = 398	Male, n = 241	p-Value
Presence of caries	294 (46%)	170 (27%)	0.3603
Poor oral hygiene	125 (20%)	75 (12%)	0.5667
Presence of diastema	76 (12%)	56 (9%)	0.2126
Presence of open bite	48 (8%)	15 (3%)	0.0134
DAI 1 (≤25): no or minimal treatment need	28 (4%)	20 (3%)	0.4761
DAI 2 (26–30): elective treatment need	34 (5%)	13 (2%)	
DAI 3 (31–35): definite treatment need	40 (6%)	23 (4%)	
DAI 4 (≥36): severe treatment need	296 (46%)	185 (29%)	
Age	20 ± 10.0	19 ± 9.0	0.1099
Frequency of missing teeth	0.9 ± 1.6	0.9 ± 1.7	0.1089
Crowding (mm)	1.2 ± 0.7	1.3 ± 0.7	0.0968
Spacing (mm)	0.3 ± 0.5	0.3 ± 0.5	0.6442
Diastema (mm)	0.3 ± 0.9	0.6 ± 1.4	0.2105
Maxillary irregularity (mm)	1.9 ± 2.3	2.2 ± 4.2	0.25
Mandibular irregularity (mm)	1.4 ± 1.8	1.6 ± 2.1	0.8766
Maxillary overjet (mm)	3.3 ± 2.8	3.7 ± 3.1	0.4152
Mandibular overjet (mm)	3.3 ± 2.8	3.5 ± 2.8	0.5008
Open bite (mm)	0.5 ± 1.7	0.2 ± 0.9	0.0164
Open bite coefficient	2.0 ± 7.1	0.8 ± 3.8	0.0165
Molar relationship (mm)	0.9 ± 0.6	1.0 ± 0.6	0.8549

The results of Table 3 showed significant findings regarding caries prevalence and oral hygiene. Caries was more prevalent in adolescents and adults compared to children, with a highly significant difference (p = 0.0001). Poor oral hygiene was also more frequent in older age groups (p = 0.0008). Open bite was slightly more common in adults (5%) compared to adolescents (4%) and children (1%), though the differences did not reach statistical significance. Additionally, missing teeth were significantly more frequent in children (p = 0.0001), while crowding and diastema were more prevalent in older age groups. No significant gender differences were found for most DAI categories. The presence of severe malocclusions (DAI ≥ 36) was higher in adolescents (35%) compared to adults (27%) and children (14%), with a statistically significant difference between children and older groups (p = 0.0318). Overall, age-related factors seemed to influence the prevalence of malocclusion traits more than gender.

**Table 3:** Association between gender with DAI esthetic and occlusal characteristics Categorical variables are presented as frequencies and percentages, while continuous variables are shown as means ± standard deviation, along with p-values to assess statistical significance between sexes. The Kruskal-Wallis’s test, Mann-Whitney U test, and chi-square test were used to assess the significance of differences between groups. DAI, Dental Aesthetic Index

Variable	Children (n = 102)	Adolescents (n = 286)	Adults (n = 251)	p-Value	Children vs adolescents (p-value)	Children vs adults (p-value)	Adolescents vs adults (p-value)
Female gender	65 (10%)	168 (26%)	165 (26%)	0.2355	-	-	-
Presence of caries	57 (9%)	203 (32%)	204 (32%)	0.0001	0.006	0.0001	0.0552
Poor oral hygiene	24 (4%)	78 (12%)	98 (15%)	0.0008	0.7444	0.0043	0.006
Presence of diastema	29 (5%)	68 (11%)	35 (6%)	0.0018	0.3556	0.0019	0.0036
Presence of open bite	5 (1%)	27 (4%)	31 (5%)	0.0784	0.1327	0.0255	0.279
DAI 1 ≤ 25 (%): no or minor treatment need	2 (1%)	22 (3%)	24 (4%)	-	-	-	-
DAI 2 26–30 (%): elective treatment	6 (1%)	20 (3%)	21 (3%)	-	-	-	-
DAI 3 31–35 (%): definite treatment need	8 (2%)	23 (4%)	32 (5%)	-	-	-	-
DAI 4 ≥ 36 (%): mandatory treatment	86 (14%)	221 (35%)	174 (27%)	0.0318	0.1324	0.0183	0.1782
Frequency of missing teeth	2.1 ± 2.5	0.6 ± 1.1	0.7 ± 1.4	0.0001	0.0001	0.0001	0.2371
Crowding (mm)	1.2 ± 0.7	1.4 ± 0.7	1.2 ± 0.7	0.0372	0.0109	0.1188	0.2386
Spacing (mm)	0.3 ± 0.5	0.3 ± 0.5	0.2 ± 0.5	0.1289	-	-	-
Diastema (mm)	0.7 ± 1.6	0.4 ± 1.0	0.3 ± 1.0	0.0021	0.3519	0.0014	0.0039
Maxillary irregularity (mm)	2.3 ± 6.1	2.3 ± 2.3	1.5 ± 2.0	0.0005	0.0236	0.8253	0.0001
Mandibular irregularity (mm)	1.1 ± 1.6	1.6 ± 1.9	1.5 ± 2.0	0.2189	-	-	-
Maxillary overjet (mm)	3.9 ± 2.9	3.7 ± 2.8	3.1 ± 3.0	0.2241	-	-	-
Mandibular overjet (mm)	3.9 ± 2.9	3.6 ± 2.9	3.0 ± 2.6	0.2544	-	-	-
Open bite (mm)	0.1 ± 0.6	0.4 ± 1.4	0.5 ± 1.7	0.0991	-	-	-
Open bite coefficient	0.4 ± 2.5	1.6 ± 5.4	2.0 ± 7.1	0.0992	-	-	-
Molar relationship (mm)	1.0 ± 0.5	0.9 ± 0.6	0.9 ± 0.6	0.3526	-	-	-

Table 4 shows the association between malocclusion severity, as measured by the DAI, and several esthetic and occlusal characteristics in a Mexican population. The most significant findings indicated that the presence of missing teeth (OR = 8.9), maxillary irregularity (OR = 8.6), and open bite (OR = 4.9) were strongly associated with more severe malocclusion (DAI 3 or 4). Additionally, alterations in maxillary overjet (OR = 6.7), mandibular overjet (OR = 6.1), and molar relationships (OR = 3.0) were also significantly correlated with higher DAI scores. Other factors, such as crowding and spacing, were present but did not show a strong association with malocclusion severity. No significant differences were observed based on gender or the presence of diastema. The analysis showed that age groups (children, adolescents, and adults) did not significantly affect the overall malocclusion severity, although more severe malocclusions were found in adolescents and adults.

**Table 4:** Association between malocclusion severity and DAI esthetic and occlusal characteristics The table displays the association between malocclusion severity (DAI 1 or 2 and DAI 3 and 4) (dependent variable) and various esthetic and occlusal characteristics (independent variables). Categorical variables, such as the presence of missing teeth or maxillary irregularities, are summarized as frequencies (%), while continuous variables, such as maxillary overjet, are presented as means ± standard deviations. The p-values indicate the statistical significance of each association. DAI, Dental Aesthetic Index

Independent variables	DAI 1 and DAI 2, n = 95	DAI 3 or DAI 4, n = 544	Total, n = 639	Odds ratio	95% CI	p-Value
Presence of missing teeth	13 (14%)	208 (38%)	221 (35%)	8.9	(7.4-13.0)	0.0001
Presence of maxillary irregularity	39 (42%)	334 (62%)	373 (58%)	8.6	(4.0-18.0)	0.0001
Presence of mandibular irregularity	37 (39%)	276 (51%)	313 (49%)	0.4	(0.9-0.19)	0.1068
Maxillary overjet alteration	10 (11%)	402 (74%)	412 (65%)	6.7	(4.2-20.0)	0.0001
Mandibular overjet alteration	22 (24%)	6 (1%)	26 (4%)	6.1	(3.0-15.0)	0.0001
Presence of open bite	9 (10%)	54 (10%)	63 (11%)	4.9	(1.4-16.0)	0.0108
Molar relationship alteration	70 (74%)	448 (83%)	518 (81%)	3	(1.5-6.2)	0.0021
Presence of diastema	11 (12%)	121 (22%)	132 (21%)	0.9	(0.9-2.0)	0.9404
Presence of crowding	84 (88%)	445 (82%)	529 (83%)	1.4	(0.5-3.0)	0.4954
Presence of spacing	17 (18%)	154 (29%)	171 (27%)	0.6	(0.1-2.0)	0.462
Female gender	62 (65%)	336 (62%)	398 (63%)	0.8	(0.4-1.6)	0.616
Children	8 (9%)	94 (17%)	-	-	-	-
Adolescents	42 (44%)	244 (45%)	-	-	-	-
Adults	45 (47%)	206 (38%)	639 (100%)	0.45	(0.15-1.3)	0.9756

## Discussion

The present study aimed to assess the prevalence of malocclusions and orthodontic treatment needs in a Mexican population using the DAI. The results demonstrated no statistically significant association between DAI scores and variables such as gender, oral hygiene, or age group. These findings are noteworthy compared to previous research and provide valuable insights into the application of the DAI in clinical settings and its implications for orthodontic treatment in Mexican populations.

Notably, our study involving a Mexican population revealed a high prevalence of severe malocclusion, with more than 50% showing a DAI score of 36 or higher, similar to findings from a Colombian cohort. This consistency suggests a significant orthodontic treatment need within these populations, potentially influenced by local genetic and environmental factors [10]. Another study on Mexican adolescents also supported a high prevalence of treatment needs, particularly due to crowding, mirroring the conditions seen in our clinical setting. This suggests that similar environmental or genetic factors might influence these populations [11]. However, this high percentage contrasts with lower percentages reported in other studies, such as 29.4% among younger Mexican schoolchildren and 26.5% in a broader age group of 16-24 years. These discrepancies may be attributed to demographic variations, socioeconomic status, and particularly, the age of participants [12,13]. It is important to consider that our study population comes from a clinical orthodontic setting, which might skew the high prevalence of severe malocclusion observed. Patients in such settings are likely more aware of their orthodontic issues and the need for treatment due to the severity of their conditions. This awareness could contribute to a higher reported need for treatment than populations in broader community-based studies [14].

Our study, which analyzed 639 patient records using the DAI, found no statistically significant differences in malocclusion severity between males and females (p=0.48). Both genders exhibited similar distributions of malocclusion, and there was no evident gender-based disparity in orthodontic treatment needs. This result aligns with the findings of Cavalcanti et al., who reported no significant gender differences in DAI scores among Brazilian adolescents. In their study, 300 adolescents were assessed, and both males and females exhibited similar prevalence and severity of malocclusion. The consistency between these studies may stem from their focus on adolescent populations, where biological factors related to growth and dental development are still in progress, and gender-based differences may not have fully manifested. Furthermore, adolescents may not yet feel the aesthetic pressures or concerns that could affect their perceptions of malocclusion severity, reducing the likelihood of gender-based differences in treatment needs [15].

In contrast, Närhi et al. found significant gender differences in a study of middle-aged adults from Finland. In this population, men had more severe malocclusions than women, but women reported a greater impact on their oral health-related quality of life (OHRQoL), particularly in the psychosocial and handicap dimensions. This suggests that while men may have more pronounced clinical malocclusions, women may be more sensitive to the psychosocial and aesthetic implications of their dental issues. The disparity between this study and the current research could be attributed to the age of the participants, as adults may be more conscious of social and aesthetic factors related to malocclusion, which could heighten the gender differences not seen in adolescent groups [16]. Similarly, Rantavuori et al. found gender differences in the association between malocclusion traits and OHRQoL in a study of Finnish adults. The findings suggest that gender plays a significant role in how malocclusion impacts quality of life, with women more affected by the psychosocial consequences of malocclusion, even when clinical severity is comparable. The difference from the present study might again be due to the adult focus of Rantavuori et al.’s research, where gender-specific societal pressures around aesthetics are more pronounced, compared to younger populations such as adolescents [17].

The current study did not find significant associations between age groups and malocclusion severity, which contrasts with findings from several previous studies. For instance, Dimberg et al. reported that malocclusion traits changed significantly across age groups, with certain traits self-correcting and others, such as deep bite, emerging during the transition from primary to permanent dentition [18]. Similarly, Balachandran and Janakiram found a higher prevalence of malocclusion in specific age groups, particularly among 13-year-old Indian children, highlighting that malocclusion severity can vary with age [19]. Lombardo et al. also noted changes in malocclusion traits such as crossbite and crowding as children grew older, even though the overall prevalence of malocclusion remained stable [20]. De Ridder et al. observed variations in the prevalence of specific malocclusion traits such as Angle's class II and III across different age groups, further supporting the idea that age influences malocclusion severity. The differences in findings between the current study and previous research can be explained by several factors. Population differences, such as variations in genetic, cultural, and environmental factors, may influence dental development and malocclusion severity [21].

The current study found a statistically significant higher prevalence of open bite malocclusion in females, consistent with previous research. Studies by Curto et al., Silvola et al., and Rantavuori et al. also reported that open bite had a greater impact on OHRQoL in women, with females showing higher Oral Health Impact Profile-14 (OHIP-14) severity scores related to psychosocial and aesthetic concerns compared to males. These findings suggest that women may be more sensitive to the aesthetic and social implications of malocclusion, particularly open bite, affecting their self-esteem and social interactions. This gender disparity in perception may explain why open bite has a more significant impact on females across various studies [17,22,23].

The current study found a higher prevalence of missing teeth in children, which was unexpected, especially given that the children's group had the smallest sample size among the three groups evaluated. This result contrasts with findings from Rakhshan and Rakhshan, who reported a lower overall prevalence of congenitally missing teeth across various ethnicities and regions, with a global mean of 6.53%. The variation in missing teeth rates may be attributed to differences in the type of missing teeth being studied (congenitally missing versus early loss of primary teeth) as well as geographical and ethnic factors. Rakhshan and Rakhshan's study emphasized regional and ethnic differences, with higher rates observed in eastern Asians and Mongoloids, but did not focus on early tooth loss in children [24]. On the other hand, Ahamed et al. reported a higher prevalence of early loss of primary teeth in Indian children, similar to the current study’s findings, with the highest occurrence among eight-year-olds. The differences in missing tooth prevalence between these studies could result from the specific populations examined, the type of missing teeth analyzed, and age-related factors influencing tooth loss [25].

The current study found a significant association between missing teeth and higher DAI scores, which is consistent with previous research on the impact of missing teeth on malocclusion. Liu et al. demonstrated that congenitally missing teeth were significantly associated with temporomandibular disorders, particularly in participants with multiple missing teeth [26]. Similarly, Cua-Benward et al. reported a higher prevalence of congenitally missing teeth in patients with class II and class III malocclusions, which can worsen occlusal discrepancies and contribute to higher malocclusion severity [27]. Moreover, Costa et al. found that tooth agenesis was associated with skeletal malocclusions, specifically noting that individuals with missing teeth had a reduced A point-nasion-B point (ANB) angle, indicating a correlation between tooth agenesis and the severity of skeletal discrepancies. These studies support the idea that missing teeth, whether congenitally absent or due to other causes, are strongly linked to increased malocclusion severity, as seen in the present study [28].

Our study identified maxillary irregularity and open bite as strong predictors of severe malocclusion - a finding consistent with previous research. Saghiri et al. highlighted the multifactorial nature of malocclusion, with genetic factors and behaviors such as mouth breathing contributing to open bite and maxillary irregularities, which, in turn, exacerbate malocclusion severity [29]. Additionally, Keyser et al. found that the severity of anterior open bite is correlated with functional issues such as speech distortions, reinforcing the current study’s conclusion that open bite not only affects dental alignment but also has broader functional and aesthetic consequences [30]. These findings emphasize the critical role of both structural irregularities and behaviors in predicting severe malocclusion.

In the current study, high ORs for maxillary and mandibular overjet alterations (OR = 6.7 and OR = 6.1, respectively) were found, indicating a strong association with severe malocclusion. This aligns with findings by Alyafrusee et al., who examined the TMJ in skeletal class I malocclusions with varying degrees of overjet and overbite. Their study showed significant changes in TMJ components, including vertical condylar positioning and condylar height, in patients with severe overjet, suggesting that overjet alterations can significantly affect jaw mechanics and may predispose individuals to temporomandibular disorders [31]. Similarly, Ghafari explored the functional role of overjet in orthodontic treatment and emphasized that overjet can mediate or aggravate jaw growth, particularly in class II and class III malocclusions, and must be carefully managed during treatment to prevent interference with mandibular movement [32].

In the current study, no strong association was found between crowding and malocclusion severity, which contrasts with the findings of other studies. Yuvashree et al. reported that mandibular arch crowding was prevalent, particularly in class I malocclusions, with nearly 46% of the patients exhibiting crowding, especially among children. Their study emphasized that crowding was more common in younger populations and had a slight female predilection [33]. Additionally, Gambardela-Tkacz et al. conducted a long-term follow-up study on incisor irregularity and found that even though mild crowding relapsed more frequently than severe crowding after treatment, both mild and severe crowding could be managed effectively in the long term with appropriate orthodontic intervention [34]. The differences in findings could be attributed to the sample composition, as the current study may have included different types of malocclusions, which could affect the association between crowding and malocclusion severity.

This study revealed a high prevalence of severe malocclusions (DAI ≥ 36) in more than 50% of participants, much higher than the 30-35% reported in other Latin American studies, potentially due to differences in access to care or genetic factors. This highlights the need for accessible orthodontic care and early intervention in Mexico, particularly through school screening programs, to reduce the need for complex treatments later. Additionally, the high prevalence of open bite malocclusion, which can lead to speech and chewing difficulties, underscores the importance of early orthodontic intervention to prevent long-term issues [35].

This study offers several strengths, including its large sample size and the use of a widely recognized and validated index (DAI) for assessing malocclusion severity. The adoption of the DAI by the WHO further underscores its value in providing reliable and comparable data across different populations. Additionally, the multivariate analysis used in this study allowed for the control of potential confounders, enhancing the robustness of the findings. However, there are also limitations to consider. The retrospective design of the study may have introduced biases related to incomplete or inaccurate clinical records, particularly regarding patient-reported oral hygiene habits. Additionally, because the study was conducted at a university-based clinic, the findings may not be generalizable to the broader Mexican population, particularly those in rural areas or with limited access to dental care. A larger, population-based study would be needed to confirm these findings across different regions and socioeconomic groups.

## Conclusions

In conclusion, this study highlights the high prevalence of severe malocclusions in a Mexican, while no significant associations were found between malocclusion severity and gender, age, or oral hygiene. The high rate of malocclusions points to the importance of public health initiatives aimed at early detection and treatment.
